# BANF1 at the crossroads of genome stability and innate immunity: emerging implications for hematologic malignancies

**DOI:** 10.3389/fimmu.2026.1990375

**Published:** 2026-10-07

**Authors:** Xia Jiang, Yihan Zhang, Dong Chen, Fenglin Li, Haihui Zhuang, Shuangyue Li, Renzhi Pei, Ying Lu

**Affiliations:** 1 Department of Hematology, The Affiliated People’s Hospital of Ningbo University, Ningbo, China; 2 Institute of Hematology, Ningbo University, Ningbo, China

**Keywords:** BANF1, cGAS-STING, clonal hematopoiesis, DNA damage response, genome stability, hematologic malignancies, nuclear envelope, replication stress

## Abstract

Genomic instability and immune escape are linked features of hematologic malignancies, but the mechanisms that allow malignant cells to tolerate persistent nuclear and genotoxic stress while avoiding damaging DNA-sensing responses remain incompletely defined. Barrier-to-autointegration factor 1 (BANF1) is a small DNA-binding protein that bridges chromatin, couples it to nuclear-envelope proteins, supports postmitotic nuclear assembly and rupture repair, modulates several DNA-damage-response pathways, and restricts productive engagement of cyclic GMP-AMP synthase (cGAS) with self-DNA. Human BANF1 variants associated with progeroid disease and motor neuronopathy, together with functional studies in solid tumors, establish organism-level and cancer relevance, but they do not establish a selective blood-cancer dependency. Here, we integrate direct BANF1 evidence with disease-specific studies of replication stress, repair dependence, and innate DNA sensing in acute myeloid leukemia, T-cell acute lymphoblastic leukemia, diffuse large B-cell lymphoma, multiple myeloma, myelodysplastic syndromes, and virus-associated lymphoid malignancies. The evidence supports a focused question: whether defined malignant hematopoietic states require BANF1 more than normal hematopoiesis, and which BANF1 function accounts for that difference. Direct hematologic evidence remains concentrated in gammaherpesvirus-associated models and concerns viral reactivation and innate signaling rather than a demonstrated lymphoma-survival dependency. We therefore distinguish direct perturbation evidence from related disease biology and from mechanistic hypotheses, emphasize that repair regulation is context dependent and that cGAS recruitment is not equivalent to cGAMP production, and critically assess survival evidence, pharmacologic tractability, and normal-cell safety. BANF1 therefore remains a mechanistically compelling candidate for disease-specific investigation in hematologic malignancies, with selectivity, signaling context, and normal-cell safety unresolved.

## 1 Introduction

Genomic instability and immune escape are interdependent features of hematologic malignancies. Leukemic, lymphomatous, and plasma-cell neoplasms often arise under sustained proliferative pressure, replication stress, chromosome-segregation errors, and altered DNA-damage-response (DDR) programs. These processes fuel clonal evolution and treatment resistance, but excessive damage also threatens nuclear integrity, exposes self-DNA, and can trigger cell death or innate immune signaling. Malignant cells must therefore preserve sufficient genome and nuclear stability to proliferate while tolerating a high burden of endogenous and treatment-induced stress.

This dependence on stress-buffering pathways is experimentally evident. T-cell acute lymphoblastic leukemia (T-ALL) can depend on CHK1 to limit replication-associated damage (1), and multiple myeloma (MM) shows an exploitable ATR dependence, including synergy between ATR inhibition and melphalan (2). Acute myeloid leukemia (AML) similarly contains replication-stress states that can be sensitized by CHK1 or ATR inhibition (3, 4). Genome stress also intersects with innate immunity: pharmacologic STING activation can produce antileukemia immune responses in AML models (5), whereas bortezomib can engage cGAS-STING-associated immunogenic responses in MM (6). In contrast, chronic cGAS-STING-NLRP3 signaling in myelodysplastic syndromes (MDS) can contribute to ineffective hematopoiesis and inflammatory injury (7, 8). Thus, the biological consequence of DNA sensing is not uniformly beneficial or harmful; it depends on disease, cell compartment, signal duration, and pathway competence.

BANF1, also called BAF and distinct from the SWI/SNF BAF complex, is relevant because it sits at the interface of these problems. BANF1 forms DNA-binding dimers, links chromatin to LEM-domain proteins and lamins, supports postmitotic nuclear assembly and nuclear-envelope repair, modulates selected repair enzymes, and restrains productive cGAS engagement with self-DNA (9–20). These functions make BANF1 more than a structural nuclear-envelope component, but they also make simplistic therapeutic assumptions unsafe: the same protein can preserve nuclear integrity, restrain PARP1 or DNA-PK activity, support MRN-associated signaling in another context, and raise the threshold for innate DNA sensing.

Human genetics strengthens the biological rationale. Homozygous BANF1 p.Ala12Thr (A12T) causes Nestor-Guillermo progeria syndrome, and patient-cell work links this variant to weakened lamin A/C engagement and mechanically fragile rupture repair (21, 22). A distinct *de novo* heterozygous p.Gly16Arg (G16R) variant has been associated with dominant motor neuronopathy and altered BANF1 conformational behavior and DNA binding (23). These variants demonstrate organism-level consequences of altered BANF1 function, but they are not evidence of leukemia predisposition and do not identify an anticancer therapeutic window.

Cancer relevance is supported by direct perturbation in solid-tumor models. BANF1 is increased in selected gastric and colorectal cancer settings, and genetic manipulation can alter growth, invasion, survival, or treatment response (24–26). Tumor-intrinsic BANF1 deletion can also enhance cGAS-STING-dependent antitumor immunity and improve the effect of PD-1 blockade in melanoma and colorectal models (27). These studies justify investigation in blood cancer, but none demonstrates that malignant hematopoietic cells depend more strongly on BANF1 than normal hematopoietic cells.

The central question for this review is therefore whether a defined malignant hematopoietic state requires BANF1 more than normal hematopoiesis does, and whether any differential requirement is structural, repair-related, or immune-mediated. To keep that question explicit, we distinguish three evidence levels throughout: direct BANF1 perturbation in the stated model; related hematologic biology without BANF1 perturbation; and a proposed mechanistic connection that remains to be tested. This is a narrative review rather than a systematic review or a new patient-cohort analysis. Accordingly, the discussion proceeds from biological significance and human disease relevance through a condensed mechanistic foundation, DNA-sensing biology, direct virus-associated hematologic evidence, candidate hematologic contexts, critical synthesis, and a single Future Directions framework. Gene symbols are italicized when they refer to genes or defined genetic lesions, whereas protein and pathway names are shown in roman type; BANF1 denotes the protein unless the gene is explicitly specified.

## 2 Mechanistic foundation of BANF1 function

### 2.1 DNA bridging, nuclear-envelope coupling, and state control

BANF1 is a small DNA-binding homodimer whose opposing DNA-binding surfaces permit cross-bridging of separate DNA duplexes (9). Structural work shows that BANF1 can engage LEM-domain proteins such as emerin while also interacting with DNA, and ternary structural studies define a lamin A/C-BANF1-emerin interface that couples chromatin to nuclear-envelope components (10, 11). More recent cryo-electron microscopy extends this architecture to nucleosome-associated BANF1-lamin A/C assemblies (12). These findings support a multivalent chromatin-nuclear-envelope scaffold rather than a single linear interaction chain.

BANF1 function is strongly state dependent. VRK1 phosphorylates nucleoplasmic and mitotic BANF1, whereas lamin-associated VRK2A regulates a nuclear-envelope-associated pool; N-terminal phosphorylation reduces DNA binding (28, 29). ANKLE2-associated PP2A and PP4C support dephosphorylation in defined contexts, and K6-linked SUMOylation promotes nuclear retention, replication competence, and nuclear integrity (30–32). Expression alone is therefore an incomplete proxy for BANF1 activity. Importantly, manipulation of a kinase or phosphatase is not equivalent to BANF1 depletion: for example, reducing VRK activity can shift BANF1 toward a more DNA-bound state rather than reproducing loss of the protein.

The structural interfaces also explain why BANF1 should not be treated as a binary on/off factor. DNA binding, LEM-domain engagement, lamin association, phosphorylation, and nuclear retention can vary independently, so the same total protein abundance can correspond to different functional states. The A12T and G16R human variants reinforce this point from different directions: altered partner engagement and altered DNA-binding behavior can both be pathogenic (22, 23). For hematologic studies, measurements of total BANF1 should therefore be accompanied, where feasible, by localization and state-sensitive readouts rather than interpreted as a direct measure of functional activity.

### 2.2 Postmitotic assembly and interphase rupture repair

The strongest human-cell evidence for a structural BANF1 requirement comes from postmitotic nuclear assembly. BANF1-mediated DNA cross-bridging organizes the anaphase chromosome mass into a single nucleus. Acute BANF1 loss during mitotic exit permits abnormal membrane penetration between chromosomes and produces fragmented or lobulated nuclear assemblies (13). This is a measured nuclear-assembly phenotype; it should not be re-labeled as evidence that blood cancers are selectively BANF1 dependent.

Single-nucleus formation is relevant beyond nuclear morphology because material excluded from the primary daughter nucleus can be packaged into micronuclei, where replication and envelope integrity are frequently abnormal. BANF1-mediated chromosome enclosure therefore acts upstream of several later stress phenotypes, including micronucleus formation and access of DNA sensors to aberrant chromatin. However, this does not mean that every defect in postmitotic enclosure produces productive cGAS signaling; the downstream sensing step remains conditional and is considered separately below.

BANF1 also functions during interphase nuclear-envelope rupture. Exposed chromatin rapidly recruits predominantly nonphosphorylated BANF1, which promotes recruitment of LEM-domain and membrane components (14). The quantitative requirement is model dependent: BANF1 loss can prevent efficient barrier restoration or prolong rupture, especially for larger defects, whereas an A12T background illustrates that simple resealing and durable repair are distinct because repaired regions can remain mechanically fragile and prone to re-rupture (14, 15, 22). LEM2/CHMP7 and ESCRT-associated studies further indicate partially overlapping closure pathways rather than a universal BANF1-LEM2-CHMP7 linear cascade (33, 34).

Mechanical rupture is relevant to both cancer and normal hematopoiesis. Constricted migration disrupts nuclei in invasive tumor cells, and strong deformation can likewise cause nuclear-envelope rupture and DNA damage in migratory immune cells (35, 36). This dual relevance is important for translation: a mechanism that destabilizes tumor nuclei may also compromise physiologically migratory leukocytes. Any future claim of tumor selectivity must therefore include normal hematopoietic and immune-cell controls rather than relying only on established cancer cell lines.

### 2.3 Context-dependent regulation of DNA repair

BANF1 participates in several DDR pathways, but the direction of its effect varies. After oxidative damage, BANF1 binds PARP1 and restrains PARP1 catalytic activity; increasing BANF1 can reduce PARylation and impair repair of oxidative lesions in the tested systems (16). In a distinct double-strand-break setting, BANF1 directly restrains DNA-PKcs. BANF1 depletion increases non-homologous end joining and reduces homologous recombination, indicating that BANF1 influences repair-pathway allocation rather than simply increasing total repair capacity (17).

BANF1 is also linked to replication competence through K6-linked SUMOylation. SUMOylation promotes nuclear retention, association with lamin A/C and PCNA, and normal DNA replication, whereas disruption of this cycle produces replication failure and abnormal nuclear architecture (32). This observation provides a mechanistic bridge between S-phase stress and later mitotic or nuclear-envelope phenotypes without establishing BANF1 as a canonical replisome component. It also helps explain why the phenotype of BANF1 perturbation may depend strongly on cell-cycle state.

A different phenotype is seen in a hepatocellular carcinoma stiffness model, in which SDC4/PKD2 signaling promotes PP2A-dependent nuclear localization of BANF1, association with MRE11, MRN-complex assembly, and ATM/CHK2 signaling (18). That pathway has not been established in marrow-resident malignancies. The apparently opposing PARP1, DNA-PK, and MRN findings therefore should not be averaged into a single ‘pro-repair’ designation. Lesion type, cell-cycle state, localization, partner engagement, and tissue context determine the observed effect. **Figure 1** and **Table 1** summarize these direct mechanisms, the measured cellular phenotype, and where hematologic extrapolation begins.

**Figure 1:**
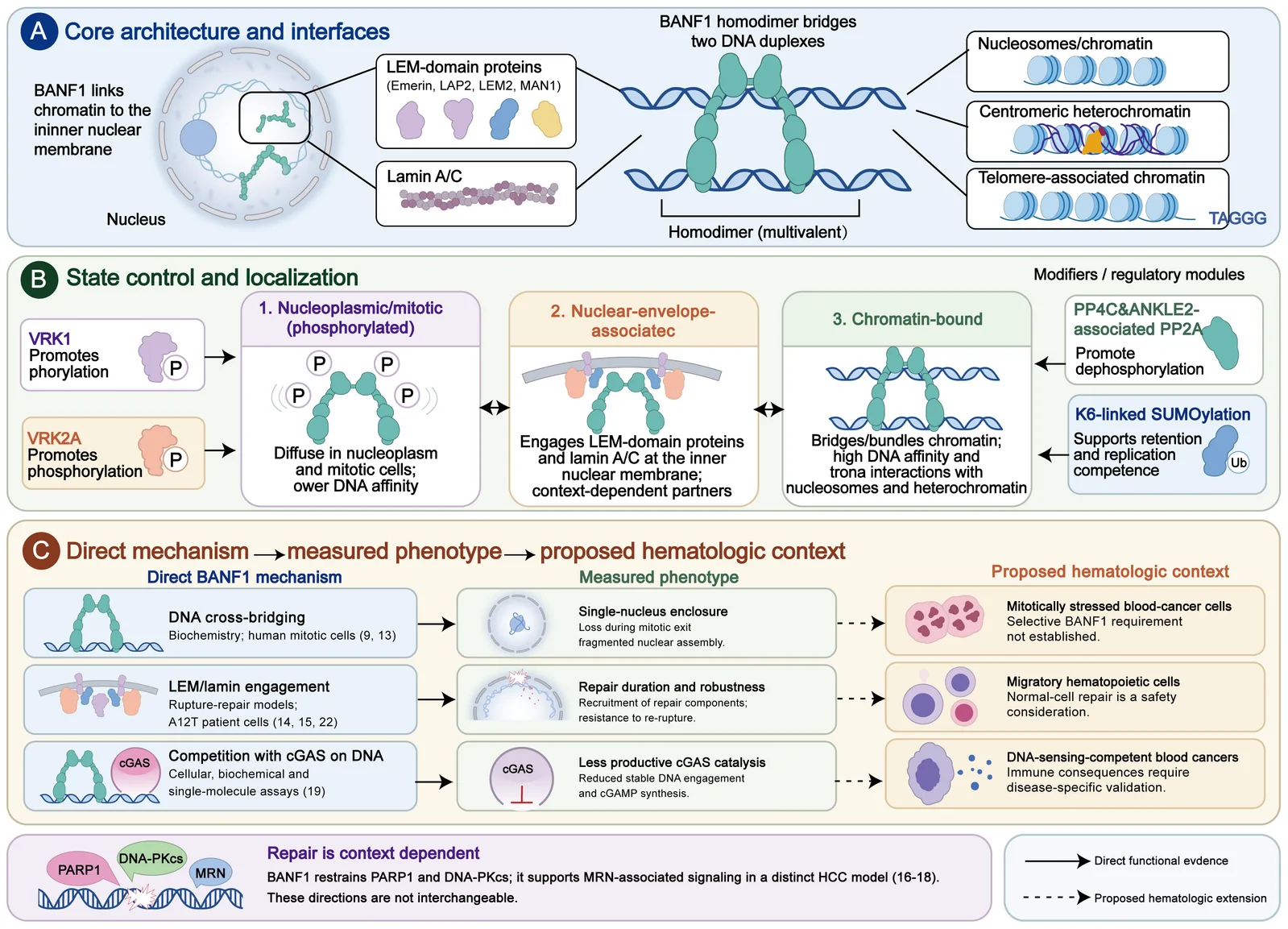
BANF1 architecture, state control, and evidence-graded functional outputs. **(A)** BANF1 forms a DNA-bridging homodimer and engages LEM-domain proteins, lamin A/C, and nucleosomal chromatin (9–12). The schematic uses simplified interaction modules rather than protein-fold or binding-geometry models. **(B)** BANF1 state is regulated by compartment-specific phosphorylation/dephosphorylation and K6-linked SUMOylation: VRK1 preferentially regulates nucleoplasmic/mitotic BANF1, VRK2A an envelope-associated pool, and PP4C and ANKLE2-associated PP2A promote dephosphorylation; SUMOylation supports nuclear retention and replication competence (28–32). **(C)** Direct mechanisms are linked first to the cellular phenotype actually measured and only then, by dashed connectors, to proposed hematologic contexts. DNA cross-bridging supports single-nucleus assembly (9, 13); LEM/lamin engagement contributes to rupture repair (14, 15, 22); and DNA-binding-competent BANF1 limits productive cGAS catalysis (19). The right-hand disease contexts are hypotheses, not established BANF1 requirements. Repair effects are context dependent: BANF1 restrains PARP1 and DNA-PKcs but supports MRN-associated signaling in a distinct HCC model (16–18). HCC, hepatocellular carcinoma; LEM, LAP2-emerin-MAN1; MRN, MRE11-RAD50-NBS1 complex.

**Table 1:** Direct BANF1 mechanisms, measured phenotypes, and limits of inference.

Mechanism/model	Directly supported finding	Boundary of the inference	References
DNA bridging and nuclear assembly; biochemical and human-cell systems	DNA cross-bridging organizes chromosome enclosure; acute mitotic loss produces fragmented nuclei.	A nuclear-assembly requirement in these systems is not evidence of selective blood-cancer dependence.	(9, 13)
LEM/lamin coupling and rupture repair; cellular and patient-derived systems	BANF1 recruits repair components; A12T weakens lamin engagement and repair robustness.	Barrier closure, repair duration, and resistance to re-rupture are distinct endpoints.	(10, 11, 14, 15, 22)
State regulation; kinase, phosphatase, and SUMO studies	Phosphorylation, dephosphorylation, and SUMOylation alter DNA engagement, localization, or replication competence.	Manipulation of a regulator is not equivalent to BANF1 depletion and may have BANF1-independent effects.	(28–32)
Repair regulation; oxidative lesions, DSBs, and HCC stiffness models	BANF1 restrains PARP1 and DNA-PKcs; nuclear BANF1 supports MRN-associated signaling in the HCC model.	Direction varies with pathway and model. These findings do not define a universal pro-repair function.	(16–18)
cGAS restriction and bridge stability; cellular and reconstituted systems	BANF1 destabilizes productive cGAS–DNA engagement and can limit bridge-associated inflammatory responses.	cGAS recruitment is not itself evidence of cGAMP synthesis; no direct BANF1-to-STING inhibitory interaction is inferred.	(19, 20, 43)

Direct evidence is restricted to the stated model and endpoint. DSB, DNA double-strand break; HCC, hepatocellular carcinoma; MRN, MRE11-RAD50-NBS1 complex. This table summarizes evidence, not proposed experiments.

## 3 BANF1 and innate DNA sensing: access is not activation

### 3.1 Direct competition with cGAS

BANF1 provides a second layer of protection against inappropriate sensing of genomic self-DNA. In cellular, purified-protein, and single-molecule experiments, DNA-binding-competent BANF1 limits cGAMP production by competing with cGAS for DNA and shortening the residence time of cGAS on DNA, thereby reducing formation of stable, catalytically productive assemblies (19). A LEM-binding-defective BANF1 mutant retains substantial immune-restraining activity, whereas a DNA-binding-defective mutant does not, placing the principal checkpoint at the substrate-sensor interface rather than at STING itself.

BANF1 can also influence the amount of DNA available for sensing. An independent study found increased basal cytosolic DNA of nuclear and mitochondrial origin and cGAS-STING-dependent signaling after BANF1 loss (20). These data support two related but separable functions: BANF1 can affect DNA compartmentalization and can reduce the probability that accessible DNA supports productive cGAS catalysis. Neither mechanism implies a direct BANF1-STING inhibitory interaction.

This distinction between access and catalysis is particularly important in cancer biology. A damaged or ruptured nucleus may expose DNA, and cGAS may even localize to that substrate, yet the biochemical threshold for cGAMP synthesis can remain unmet. BANF1 therefore acts most defensibly as a regulator of productive sensor engagement rather than as a generic suppressor of all DNA-triggered inflammation. That framing also avoids conflating direct cGAS competition with downstream STING biology, which can differ among cell types and malignancies.

### 3.2 Micronuclei, chromatin bridges, and context-dependent signaling

Micronuclear rupture illustrates why access and activation must be separated. Early studies connected micronuclei with cGAS recruitment and inflammatory signaling (37, 38), but later work showed that micronuclei generated by irradiation, replication stress, or chromosome-segregation errors can recruit cGAS without detectable cGAMP production or downstream STING-TBK1-IRF3 activation (39, 40). A single-cell cGAMP reporter further showed that genotoxic-stress-induced activation is uncommon and that cGAS-positive micronuclei are poor surrogates for productive sensing (41). The appropriate conclusion is not that micronuclei are never immunostimulatory, but that cGAS localization alone is insufficient evidence of catalytic activation.

Chromatin bridges represent a non-equivalent route. Bridge-associated cGAS can correlate with signaling after selected drug-induced mitotic errors (42). BANF1 stabilizes chromatin bridges, and its depletion can increase bridge breakage and cGAS-recruiting remnants; combined BANF1 loss and MPS1 inhibition strengthens cGAS-STING-dependent inflammatory transcription in the tested models (43). These findings support a structural checkpoint in specific mitotic-error settings, not a universal stress-sensing pathway upstream of BANF1.

Taken together, the micronucleus and chromatin-bridge literature argues against a single structural surrogate for innate activation. Micronuclei, bridge remnants, nuclear ruptures, and unresolved replication lesions differ in chromatin organization, mechanical state, nuclease exposure, and duration. These variables can change both the probability of cGAS binding and the probability that bound cGAS becomes catalytically productive. Future BANF1 studies should therefore classify the abnormal DNA substrate rather than pooling all forms of ‘cytosolic’ or ‘exposed’ DNA into one category.

The biological consequence of overcoming this checkpoint is also context dependent. Tumor-intrinsic BANF1 loss enhances cGAS-STING-dependent antitumor immunity in selected solid-tumor models (27), whereas hematologic diseases show both potentially beneficial and potentially injurious STING biology. **Figure 2** therefore separates mechanical, mitotic, and genotoxic stress routes; DNA access; cGAS recruitment; productive cGAMP synthesis; downstream signaling; and the final biological outcome.

**Figure 2:**
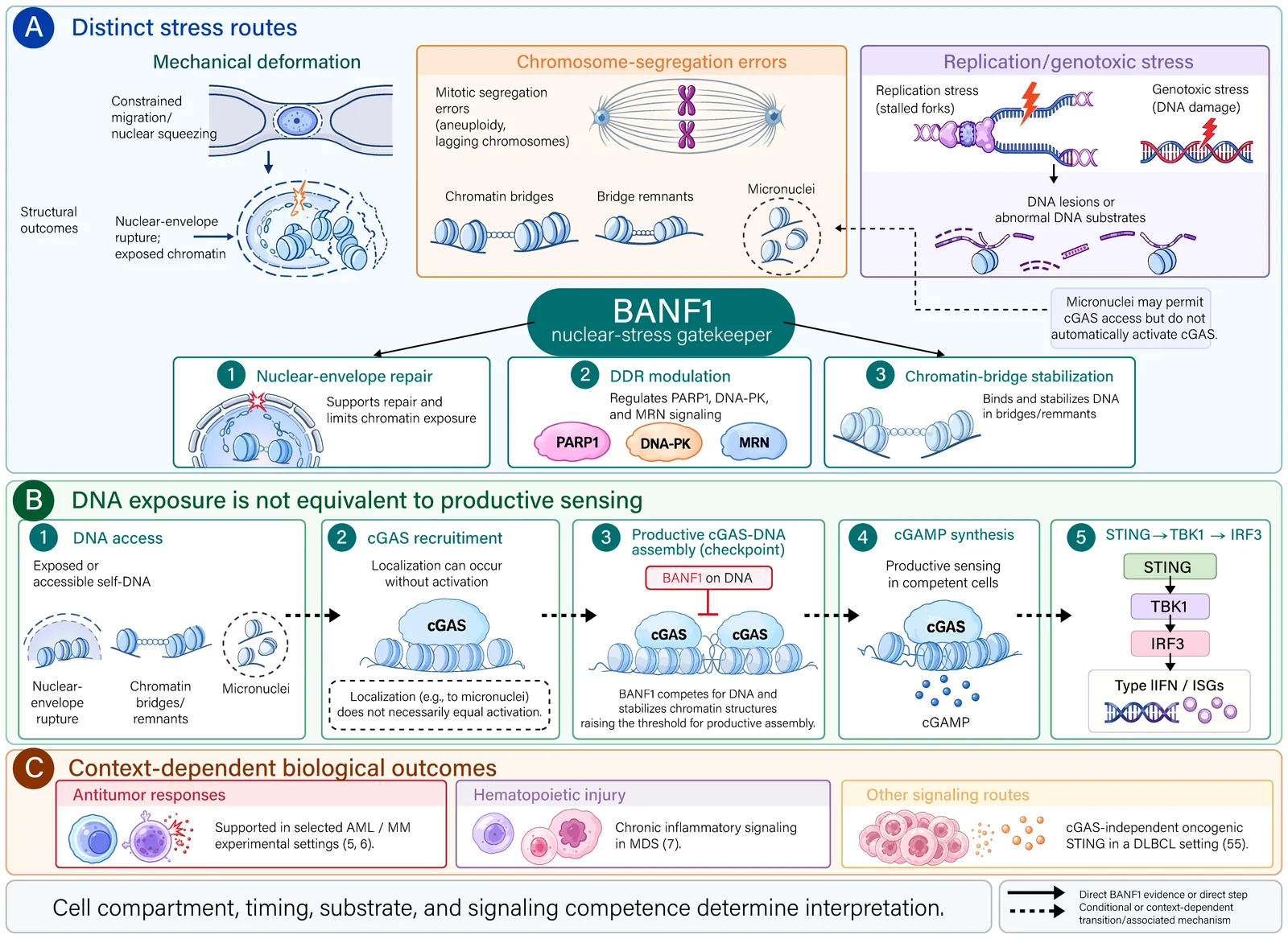
Distinct stress routes and the evidence required for productive DNA sensing. **(A)** Mechanical deformation, chromosome-segregation errors, and replication/genotoxic stress are depicted as distinct routes with non-equivalent structural consequences; their juxtaposition does not define BANF1 as a common upstream stress sensor. The BANF1 roles shown below are restricted to rupture repair, context-dependent DDR modulation, and chromatin-bridge stabilization supported in the cited systems (14–18, 43). **(B)** DNA access, cGAS recruitment, productive cGAS-DNA assembly, cGAMP synthesis, and downstream STING-TBK1-IRF3 signaling are separated conceptually. BANF1 restraint is placed at productive cGAS engagement, supported by DNA competition and cGAMP measurements (19); conditional transitions are dashed, and micronuclear or bridge localization alone does not prove catalysis (39–42). **(C)** Biological outcome is separate from pathway activation and may include antitumor responses, inflammatory hematopoietic injury, or other context-dependent signaling (5–7, 55). AML, acute myeloid leukemia; cGAMP, cyclic GMP-AMP; DLBCL, diffuse large B-cell lymphoma; DDR, DNA-damage response; IRF3, interferon regulatory factor 3; MDS, myelodysplastic syndromes; MM, multiple myeloma; TBK1, TANK-binding kinase 1.

## 4 Direct hematologic evidence: virus-associated lymphoid models

Gammaherpesvirus studies provide the most direct hematologic bridge in the literature reviewed here, but the experimental endpoint must be preserved. Broussard and colleagues showed that BANF1 depletion reduces Kaposi sarcoma-associated herpesvirus (KSHV) lytic gene expression and infectious output while increasing innate antiviral signaling (44). Their models included TREx-BCBL1-RTA cells derived from primary effusion lymphoma (PEL), as well as nonhematologic KSHV systems. Mechanistic experiments across the study implicated the cGAS-STING-IRF3 axis. These data establish a BANF1 role in viral reactivation and innate signaling; they do not establish that PEL cells require BANF1 for survival.

The same report also examined EBV-positive Akata-BX1 Burkitt lymphoma cells. BANF1 depletion increased interferon-response transcripts and reduced viral reactivation and infectious output (44). This broadens the direct hematologic-derived evidence beyond one PEL model, but it does not change the endpoint: antiviral effects are not synonymous with selective killing of malignant B cells.

Separate PEL experiments directly manipulated STING rather than BANF1. STING activation with diABZI restricted lytic reactivation and reduced growth more strongly in STING-responsive BCBL1 cells than in STING-low BC3 cells, while inhibition and re-expression experiments supported a growth-restraining role for STING in the tested settings (45). These results make downstream pathway competence relevant to any future BANF1 experiment, but they cannot be used as proof that BANF1 loss itself suppresses PEL growth. **Figure 3** deliberately places direct BANF1 viral-lifecycle data and separate STING growth data in different evidence boxes, with the proposed link to malignant-cell fitness marked as untested.

**Figure 3:**
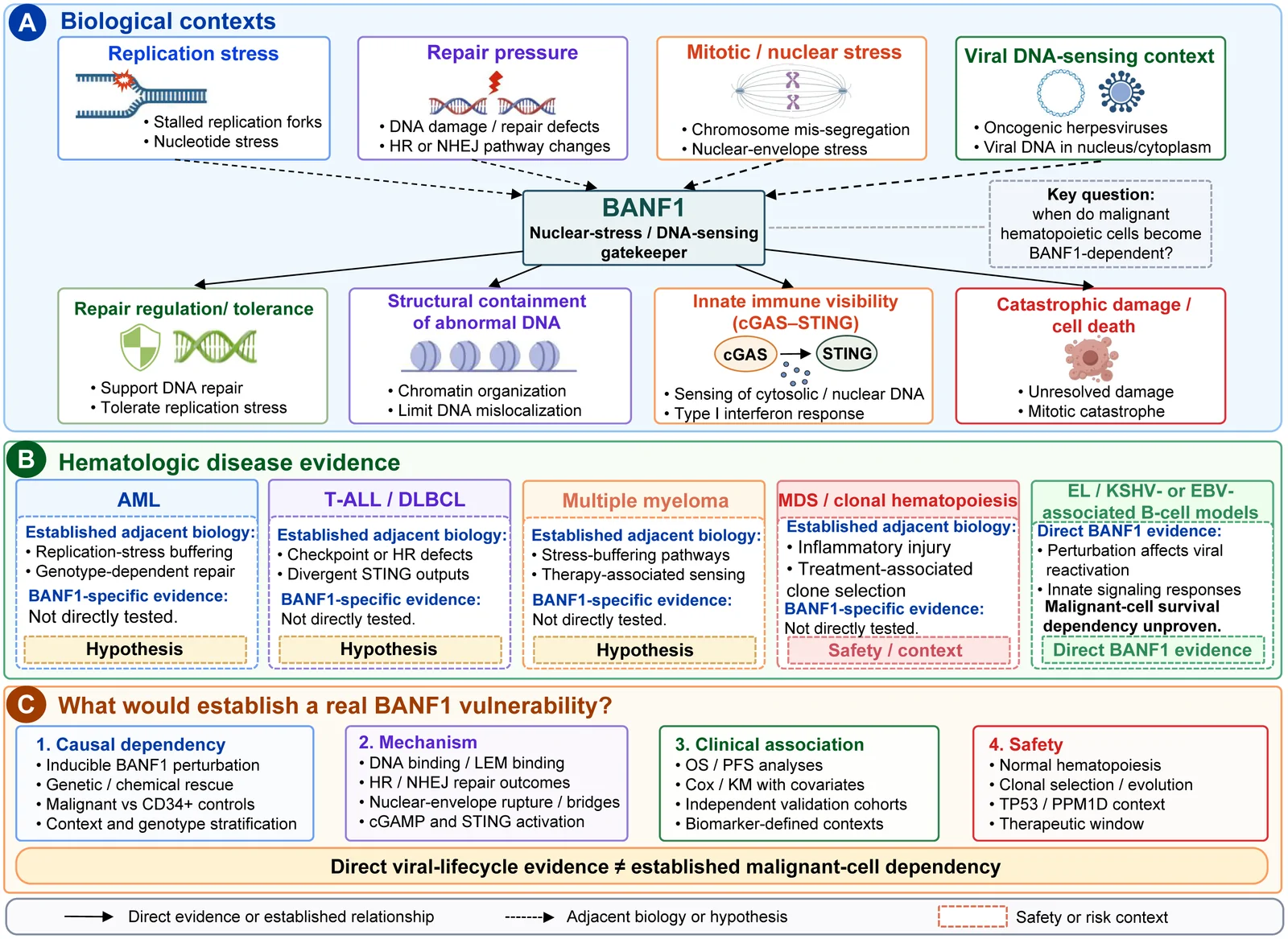
Evidence-graded roadmap for testing BANF1 in hematologic malignancies. **(A)** Replication stress, repair pressure, mitotic/nuclear stress, and viral DNA-sensing contexts define biological settings in which BANF1 may influence repair regulation, structural containment, innate DNA sensing, or stress tolerance. The dashed connectors to BANF1 and the key-question box indicate biological rationale rather than established hematologic dependency; the lower outputs are alternative conceptual consequences and should not be interpreted as a uniform direction of effect. **(B)** AML, T-ALL/DLBCL, MM, and MDS/clonal hematopoiesis are supported mainly by adjacent disease biology; BANF1-specific causality has not been established in the cited disease studies. By contrast, direct BANF1 perturbation in PEL-derived TREx-BCBL1-RTA and EBV-positive Akata-BX1 cells affects viral reactivation and innate signaling (44), while PEL growth effects derive from a separate STING study (45). Malignant-cell survival dependency remains unproven. **(C)** A claim of selective BANF1 vulnerability would require convergent evidence of causal selectivity, mechanism, clinical association, and safety, as detailed in **Table 3**. Dashed connectors denote adjacent biology or hypotheses, and red-highlighted elements denote safety/risk contexts. AML, acute myeloid leukemia; DLBCL, diffuse large B-cell lymphoma; EBV, Epstein-Barr virus; KSHV, Kaposi sarcoma-associated herpesvirus; MDS, myelodysplastic syndromes; MM, multiple myeloma; PEL, primary effusion lymphoma; T-ALL, T-cell acute lymphoblastic leukemia.

## 5 Candidate hematologic contexts

Outside virus-associated models, the hematologic literature mainly identifies stress and signaling states in which BANF1 should be tested rather than demonstrating a BANF1 requirement. **Table 2** summarizes these boundaries. Molecularly defined states are more informative than ranking entire malignancies by presumed BANF1 dependence, because even within a single diagnosis repair competence and innate signaling can differ substantially.

**Table 2:** Hematologic evidence map: direct BANF1 findings versus candidate contexts.

Disease/context	Evidence actually established	What is not established for BANF1	References
KSHV-positive PEL; EBV-positive Akata-BX1 cells	Direct BANF1 perturbation alters viral reactivation and innate signaling in hematologic-derived models.	Selective malignant-cell survival dependence or antitumor benefit from BANF1 loss. Separate STING studies address PEL growth.	(44, 45)
AML	CHK1/ATR stress buffering; genotype-specific fork and HR/PARP biology.	A BANF1-specific mechanism or validated BANF1–OS/PFS association in these cited studies.	(3, 4, 46, 47)
T-ALL and HR-deficient lymphoid states	CHK1 dependence; *EZH2*-loss-associated replication stress; *LMO2*-associated HR deficiency.	The direction or selectivity of BANF1 perturbation in these states.	(1, 51, 52)
DLBCL	DDR-active subsets; STING-linked inflammatory death or cGAS-independent oncogenic signaling in different models.	A single BANF1–STING mechanism across DLBCL or uniformly beneficial immune activation.	(53–55)
MM	ATR and helicase stress buffering; inducible HR deficiency; therapy-associated cGAS-STING responses.	Direct BANF1 survival dependence or predictable synergy with PARP or checkpoint inhibition.	(2, 6, 56–59)
MDS/clonal hematopoiesis	Inflammatory injury and therapy-driven selection of particular mutant hematopoietic clones.	Benefit from BANF1 inhibition, or evidence that BANF1 perturbation itself selects those clones.	(7, 8, 48–50)

Evidence boundaries refer to the studies cited in this review, not an exhaustive absence claim. Gene-related phrases denote defined genetic states; named pathway proteins are in roman type. AML, acute myeloid leukemia; DDR, DNA-damage response; DLBCL, diffuse large B-cell lymphoma; HR, homologous recombination; MM, multiple myeloma; MDS, myelodysplastic syndromes; OS, overall survival; PEL, primary effusion lymphoma; PFS, progression-free survival; T-ALL, T-cell acute lymphoblastic leukemia.

### 5.1 Myeloid malignancies: AML, MDS, and clonal hematopoiesis

AML illustrates how replication-stress buffering can become genotype and treatment dependent. High CHK1 activity can support survival during cytarabine stress, and CHK1 or ATR inhibition can weaken this tolerance (3, 4). Leukemia-associated *DNMT3A* mutations can impair PARP1 recruitment at stalled forks (46), while *RUNX1::RUNX1T1*- or *PML::RARA*-driven models provide another setting in which altered homologous-recombination programs confer PARP-inhibitor sensitivity (47). These findings define repair states in which BANF1 could matter, but they do not show that BANF1 expression predicts AML outcome or that all AML is repair deficient.

BANF1 is mechanistically positioned to influence how unresolved replication lesions are handled because it affects PARP1, DNA-PK, nuclear assembly, and access of cGAS to abnormal DNA (13, 16, 17, 19). AML therefore provides a rational context for testing whether partial BANF1 perturbation converts replication stress into mitotic/nuclear failure or productive DNA sensing. Importantly, this remains a proposed experiment. The antileukemia effects of direct STING activation in AML (5) cannot be assumed to predict the consequence of BANF1 loss.

The AML examples also illustrate why a disease-wide BANF1 expression comparison would be difficult to interpret. Replication stress may arise from treatment, oncogenic transcriptional programs, or defective fork protection, while homologous-recombination competence varies across molecular subtypes (3, 4, 46, 47). A BANF1 phenotype could therefore emerge only in a subset defined by stress state and repair configuration. Likewise, antileukemia STING activation in preclinical AML can involve immune-cell responses rather than a simple tumor-cell-autonomous pathway (5). These features favor genotype- and compartment-resolved experiments over a single AML-wide dependency claim.

MDS provides the opposite caution. cGAS-STING-NLRP3 signaling and inflammasome-associated pyroptosis can contribute to ineffective hematopoiesis (7, 8). Independently, cytotoxic therapy can favor pre-existing *PPM1D*- or *TP53*-mutant hematopoietic clones (48–50). Neither observation directly implicates BANF1, but both constrain interpretation: a perturbation that increases inflammation or genome stress could damage residual hematopoiesis or change clone fitness without producing an immediate fall in malignant-cell viability. MDS and clonal hematopoiesis should therefore be treated as safety and direction-of-effect contexts rather than as established indications for BANF1 inhibition.

### 5.2 Lymphoid and plasma-cell malignancies: T-ALL, DLBCL, and multiple myeloma

T-ALL provides an experimentally established checkpoint-addiction context. Baseline CHK1 dependence is supported in T-ALL models with high replication stress (1), and loss of *EZH2* can increase MYCN-linked replication stress and sensitivity to CHK1 inhibition (51). LMO2-positive T-ALL and DLBCL can also display homologous-recombination deficiency and PARP-inhibitor sensitivity (52). BANF1-dependent regulation of DNA-PK creates a plausible connection, but the direction is uncertain: altered NHEJ use could be protective or harmful depending on the pre-existing repair defect. The established CHK1 or PARP vulnerability must therefore not be re-labeled as a BANF1 vulnerability.

DLBCL further demonstrates why DNA-sensing pathways cannot be collapsed into a single model. Some DLBCL subsets show constitutive DDR activation and MYC-associated stress (53). In one setting, *SAMHD1* deficiency engages STING-associated PANoptosis (54); in another, extrachromosomal circular DNA supports oncogenic STING signaling through a cGAS-independent route (55). Because BANF1 directly restrains cGAS rather than STING, it cannot automatically explain or reverse a cGAS-independent STING program. DLBCL is therefore a strong test of mechanism specificity, not evidence that increasing STING signaling is uniformly beneficial.

Within DLBCL, the coexistence of HR-deficient states, constitutive DDR activation, and divergent STING outputs creates a useful stress test for the BANF1 model. A coherent result would require showing that BANF1 perturbation changes a defined repair or sensing phenotype in the expected molecular subgroup and that rescue or pathway epistasis reverses that effect. Without such evidence, correlations with LMO2, MYC-associated stress, or STING activity remain neighboring biology rather than a BANF1 mechanism (52–55).

MM contains several established stress-buffering dependencies. ATR inhibition can enhance melphalan activity, proteasome inhibition can induce a functional homologous-recombination-deficient state, and RECQ1 or BLM perturbation can compromise replication-stress survival (2, 56–58). Bortezomib and PARP1 trapping with talazoparib have also been linked to cGAS-STING-associated immunogenic responses in MM models (6, 59). These studies make MM a particularly informative setting in which to test whether BANF1 links lesion handling to DNA sensing. However, no cited MM study here directly establishes a BANF1 survival dependency, and BANF1’s negative regulation of PARP1 means that combination effects cannot be inferred simply from the label ‘DNA-repair protein.’

MM presents a related but distinct opportunity because several stress-buffering modules have already been functionally validated. ATR dependence, inducible BRCAness, RECQ1/BLM-associated replication-stress tolerance, and therapy-linked cGAS-STING responses are not interchangeable phenomena (2, 6, 56–59). A BANF1 experiment that measures only viability would therefore be insufficiently informative. Replication-stress markers, repair-pathway allocation, direct cGAMP production, and immune consequences should be assessed together so that a structural stress phenotype is not mistaken for an immune mechanism, or vice versa.

## 6 Critical synthesis: clinical evidence, prioritization, and translational boundaries

The clinical evidence remains weaker than the mechanistic evidence. Published gastric-cancer work reports associations between BANF1 expression and survival alongside separate functional experiments (24), but such solid-tumor associations cannot be transferred directly to leukemia, lymphoma, or myeloma. In the hematologic studies reviewed here, survival or treatment-response associations concern factors such as CHK1 or disease-specific stress states rather than a validated BANF1 prognostic model (3, 53). The available evidence therefore does not establish BANF1 as an independent predictor of overall survival (OS) or progression-free survival (PFS) in blood cancer.

Disease-specific survival analyses could add clinical context, but only when the available clinical annotation is adequate. Expression-survival association, functional requirement, and treatment-predictive utility are different claims. Even a reproducible BANF1-OS association could reflect proliferation, lineage, malignant-cell fraction, or treatment distribution rather than target dependence. Consistent with REMARK principles and curated clinical-outcome resources (60, 61), a rigorous future analysis should use separate disease cohorts, prespecified endpoint definitions and censoring rules, continuous-expression Cox models, Kaplan-Meier displays for prespecified groups, numbers at risk, event counts, proportional-hazards checks, clinically available covariate adjustment, and independent validation. PFS, event-free survival, and progression-free interval should not be treated as interchangeable endpoints.

BANF1 merits focused mechanistic investigation not because it has been shown to outperform ATR, CHK1, PARP1, lamins, or other candidate targets, but because it links several otherwise distinct stress-control processes. Its distinguishing rationale is that direct DNA bridging, nuclear-envelope repair, repair-enzyme regulation, and competition with cGAS are supported within one protein’s functional repertoire (13–19). This breadth creates a unique opportunity to ask whether structural containment and sensor access jointly determine the fate of stressed malignant cells. It also creates risk: without a demonstrated normal-cell window, mechanistic breadth could be a liability rather than an advantage.

Three controversies summarize the field. First, BANF1 can restrain PARP1 and DNA-PK yet support MRN-associated repair in another setting, so ‘pro-repair’ versus ‘anti-repair’ is the wrong binary; pathway, lesion, and localization matter (16–18). Second, acute STING activation can support antileukemia or antimyeloma responses, whereas chronic inflammatory signaling can damage MDS hematopoiesis and cGAS-independent STING signaling can be oncogenic in a DLBCL setting (5–7, 55). Third, BANF1 abundance is not equivalent to BANF1 state because phosphorylation, localization, and partner engagement alter function (28–32). A useful hematologic model must resolve these context dependencies rather than average them into one direction of effect.

Chemical tractability is similarly preliminary. Obtusilactone B and brazilin bind BAF and perturb BANF1 phosphorylation or nuclear-envelope reassembly, while biochemical assays establish the feasibility of screening interference with BANF1-dependent DNA clustering (62–64). These studies show proof of principle, not a clinically validated or selective BANF1 inhibitor. Modulating VRK1/VRK2A, PP4C, or ANKLE2-PP2A should likewise be framed as experimental manipulation of BANF1 state rather than as BANF1-directed therapy, because each regulator has BANF1-independent functions and can move BANF1 toward different biochemical states.

The main limitations of the current evidence base are therefore the small number of direct hematologic-derived BANF1 studies, heterogeneous endpoints, limited malignant-versus-normal comparisons, incomplete knowledge of pathway competence across disease subtypes, and the absence of a new patient-level outcome analysis in this narrative review. These limitations argue for narrower claims and better experiments rather than for abandoning the hypothesis.

## 7 Future directions

Future work should resolve a small number of decisive questions rather than reproduce disease-specific wish lists. **Table 3** consolidates the proposed work into one experimental matrix. The first requirement is causal selectivity: inducible BANF1 depletion or degradation should be compared across molecularly defined AML, T-ALL, DLBCL, MM, and virus-associated B-cell models together with normal CD34+ hematopoietic stem/progenitor cells and mature immune controls. Independent reagents, perturbation-depth matching, and wild-type rescue are essential to distinguish a true BANF1 requirement from nonspecific toxicity.

**Table 3:** One integrated experimental matrix for future BANF1 validation.

Decision to resolve	Proposed design/essential controls	Decisive interpretation
1. Disease-selective causal requirement	Compare inducible BANF1 depletion or degradation across molecularly defined AML, T-ALL, DLBCL, MM, and virus-associated B-cell models with normal CD34+ hematopoietic stem/progenitor cells and mature immune controls. Use independent reagents, perturbation-depth matching, and wild-type rescue; measure growth, clonogenicity, differentiation, and recovery.	A rescued, preferential malignant-cell defect supports selective requirement. Similar normal-cell injury argues against a useful window. Expression alone is not the selection criterion.
2. Structural versus repair mechanism	Use separation-of-function rescue with validated DNA-binding or LEM-binding mutants. Match cell-cycle state; quantify replication stress, HR/NHEJ reporters, PARP1 and DNA-PK activity, chromosome bridges, micronuclei, and rupture duration. Pair perturbations with pathway-specific epistasis rather than assuming drug synergy.	A pathway-specific rescue or epistasis result identifies the causal contribution. A rise in DNA-damage markers alone does not distinguish defective repair from altered cell cycling.
3. Productive DNA sensing and the responding cell	Combine direct cGAMP quantification with an orthogonal single-cell reporter, STING/TBK1/IRF3 signaling, and interferon readouts. Test cGAS and STING loss separately; distinguish malignant, immune, and progenitor compartments in mixed-cell or immunocompetent systems. In virus-associated models, measure viral output separately from malignant-cell fitness.	Loss of the relevant pathway should remove the attributed immune effect. cGAS localization or STING phosphorylation alone is insufficient. Antiviral activity does not establish lymphoma killing.
4. Clinical association, not assumed clinical utility	Use disease-specific public transcriptomic/genomic cohorts with matched follow-up; prespecify eligibility, endpoint origin, censoring, treatment context, and missing-data handling. Analyze continuous BANF1 expression with Cox models; use prespecified groups for Kaplan–Meier display, report numbers at risk, events, hazard ratios and confidence intervals, and assess proportional hazards. Adjust for available age, subtype/genotype, treatment, and cell-composition covariates; control multiplicity and validate in an independent cohort. Analyze OS and PFS separately only when their event definitions are available.	A replicated adjusted association supports prognosis, not BANF1 dependence or treatment prediction. Do not pool different malignancies or substitute PFI/EFS for PFS. Sparse-event or incompletely annotated cohorts should not support a definitive claim.
5. Chemical specificity and sustained safety	For direct ligands or state modulators, establish intracellular target engagement and compare with matched genetic perturbation and relevant rescue. Separate VRK/phosphatase effects from BANF1-mediated effects. Extend transient exposure studies to hematopoietic recovery and longitudinal, mutation-stratified clone tracking, including *TP53* and *PPM1D* contexts.	Activity without target engagement is not selective BANF1 pharmacology. Preserved short-term viability does not exclude impaired hematopoietic recovery or adverse clonal selection.

All entries are proposed studies; none is presented as newly performed in this review. The matrix integrates the mechanistic evidence in **Tables 1**, **2** and reporting principles for clinical-outcome work (60, 61). EFS, event-free survival; PFI, progression-free interval. Separation-of-function mutants require validation for expression, localization, and the intended biochemical defect before interpretation.

Second, structural and immune mechanisms must be separated. Validated separation-of-function rescue can test DNA-binding and LEM-binding contributions, while cell-cycle-matched measurements of replication stress, HR/NHEJ, PARP1/DNA-PK activity, chromosome bridges, micronuclei, and rupture duration can define the structural/repair component. Direct cGAMP quantification should be combined with an orthogonal single-cell reporter and downstream STING/TBK1/IRF3 measurements; cGAS or STING loss should be used separately to establish pathway dependence. In virus-associated models, viral output and malignant-cell fitness must be measured as different endpoints.

Third, clinical association should proceed in parallel but should not substitute for perturbation evidence. Disease-specific public transcriptomic/genomic cohorts with matched follow-up can test whether BANF1 expression or a defined functional-state surrogate associates with OS or PFS, but any association should be described as prognostic until treatment-interaction or functional data justify a stronger interpretation. Null results are also informative because a mechanistically important protein need not show a strong between-patient expression-outcome gradient.

An interpretable negative result is also important. If matched BANF1 perturbation produces similar injury in malignant cells and normal HSPCs, that would argue against a useful selective window even if the mechanism is biologically interesting. Conversely, lack of interferon signaling in a STING-deficient model would not exclude a structural BANF1 requirement. The experimental matrix is therefore designed to distinguish target dependence, pathway competence, and safety rather than to force all disease models toward a single positive conclusion.

Finally, safety requires more than short-term viability. BANF1 supports fundamental nuclear functions, normal migratory immune cells can experience nuclear rupture, and treatment can reshape the fitness of pre-existing hematopoietic clones (35, 36, 48–50). Preclinical development should therefore include hematopoietic recovery, differentiation, colony formation, and longitudinal mutation-stratified clone tracking. Until disease-selective causality, target engagement, and an acceptable normal-cell window are demonstrated together, BANF1 should remain an experimental research candidate rather than a validated hematologic therapeutic target.

## 8 Conclusions

BANF1 lies at a biologically coherent intersection of chromatin organization, nuclear-envelope integrity, DNA-repair regulation, and innate DNA sensing. DNA cross-bridging supports postmitotic single-nucleus assembly; LEM/lamin interactions contribute to rupture repair; post-translational state regulates localization and DNA engagement; and BANF1 directly raises the threshold for productive cGAS activation on self-DNA (9–19, 28–32). At the same time, the PARP1, DNA-PK, and MRN literature shows that BANF1 cannot be reduced to a uniformly pro-repair factor.

For hematologic malignancies, disease-selective BANF1 dependency remains a hypothesis. AML, T-ALL, DLBCL, and MM provide well-defined replication-stress, checkpoint, repair, or DNA-sensing contexts in which BANF1 can be tested, while MDS and clonal hematopoiesis highlight the risks of inflammatory injury and adverse clone selection. Virus-associated B-cell models provide the most direct hematologic-derived BANF1 evidence reviewed here, but that evidence concerns viral reactivation and innate signaling rather than an established lymphoma-survival mechanism (44, 45). The next step is therefore not broader cataloguing, but disease-selective causal testing with normal-cell controls, mechanistic rescue, direct cGAMP measurement, and clinically rigorous outcome analysis. Such evidence will determine whether BANF1 remains an informative mechanistic node or can be advanced as a credible candidate therapeutic vulnerability.
